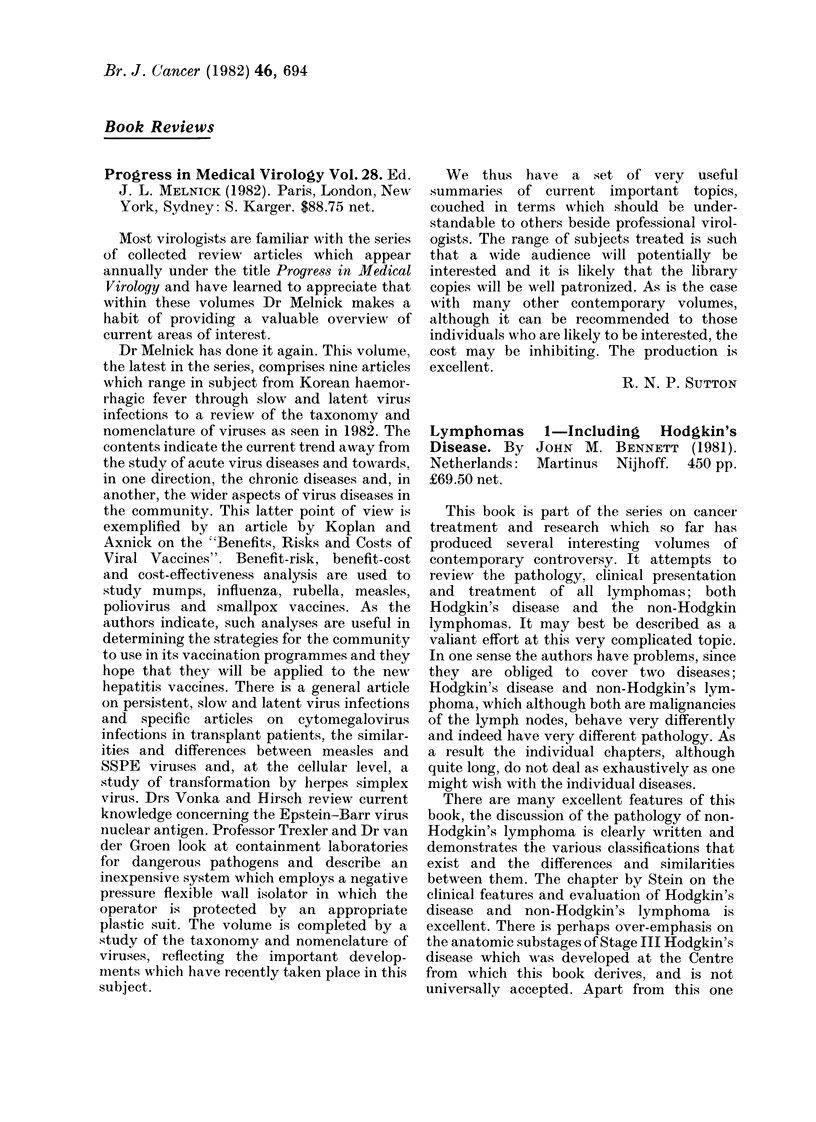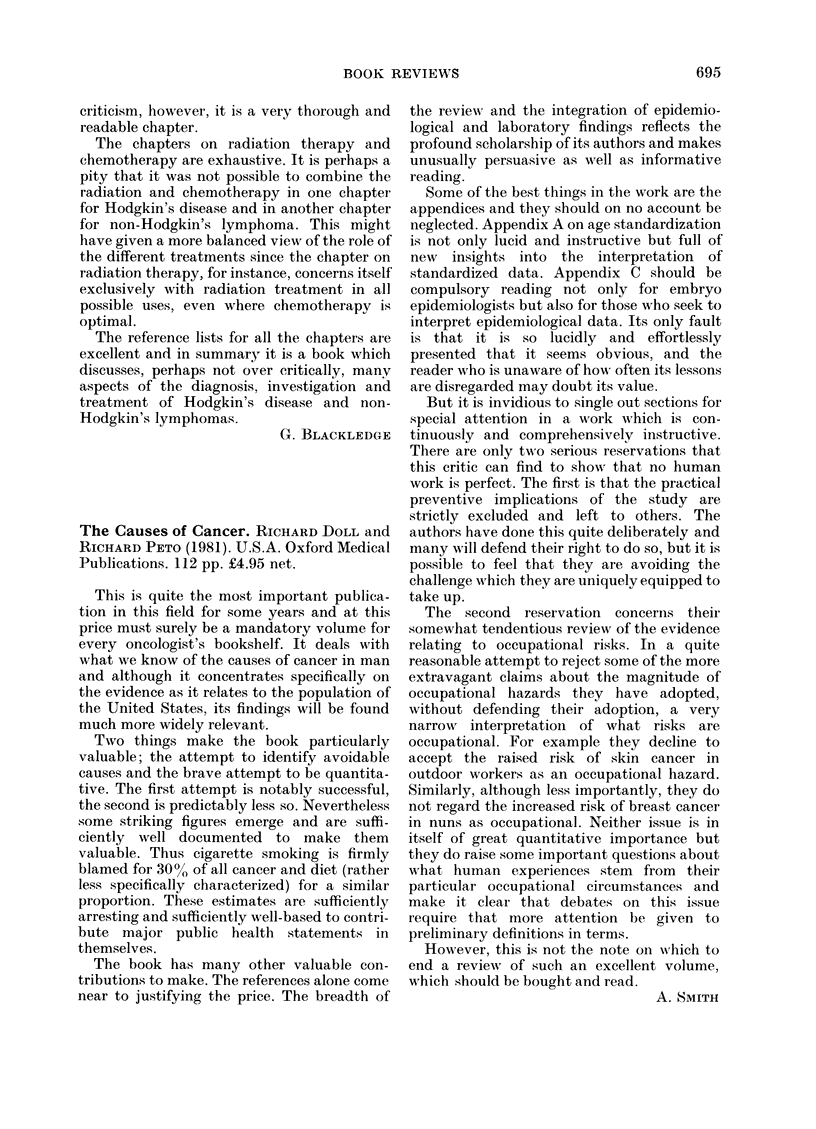# Lymphomas 1—Including Hodgkin's Disease

**Published:** 1982-10

**Authors:** G. Blackledge


**Lymphomas 1—Including Hodgkin's Disease.** By JOHN M. BENNETT (1981). Netherlands: Martinus Nijhoff. 450 pp. £69.50 net.

This book is part of the series on cancer treatment and research which so far has produced several interesting volumes of contemporary controversy. It attempts to review the pathology, clinical presentation and treatment of all lymphomas; both Hodgkin's disease and the non-Hodgkin lymphomas. It may best be described as a valiant effort at this very complicated topic. In one sense the authors have problems, since they are obliged to cover two diseases; Hodgkin's disease and non-Hodgkin's lymphoma, which although both are malignancies of the lymph nodes, behave very differently and indeed have very different pathology. As a result the individual chapters, although quite long, do not deal as exhaustively as one might wish with the individual diseases.

There are many excellent features of this book, the discussion of the pathology of non-Hodgkin's lymphoma is clearly written and demonstrates the various classifications that exist and the differences and similarities between them. The chapter by Stein on the clinical features and evaluation of Hodgkin's disease and non-Hodgkin's lymphoma is excellent. There is perhaps over-emphasis on the anatomic substages of Stage III Hodgkin's disease which was developed at the Centre from which this book derives, and is not universally accepted. Apart from this one criticism, however, it is a very thorough and readable chapter.

The chapters on radiation therapy and chemotherapy are exhaustive. It is perhaps a pity that it was not possible to combine the radiation and chemotherapy in one chapter for Hodgkin's disease and in another chapter for non-Hodgkin's lymphoma. This might have given a more balanced view of the role of the different treatments since the chapter on radiation therapy, for instance, concerns itself exclusively with radiation treatment in all possible uses, even where chemotherapy is optimal.

The reference lists for all the chapters are excellent and in summary it is a book which discusses, perhaps not over critically, many aspects of the diagnosis, investigation and treatment of Hodgkin's disease and non-Hodgkin's lymphomas.

G. BLACKLEDGE